# Does Collaboration between General Practitioners and Pharmacists Improve Risk Factors for Cardiovascular Disease and Diabetes? A Systematic Review and Meta-Analysis

**DOI:** 10.5334/gh.1184

**Published:** 2023-02-23

**Authors:** Kanika Chaudhri, Gabriella Caleres, Samantha Saunders, Peter Michail, Gian Luca Di Tanna, Thomas Lung, Hueiming Liu, Rohina Joshi

**Affiliations:** 1 The George Institute for Global Health, UNSW, Sydney, Australia; 2 Department of Clinical Sciences, Center for Primary Health Care Research, Lund University, Malmö, Sweden; 3 Gosford Hospital, Central Coast Local Health District, Australia; 4 General Surgery, St Vincent’s Hospital, Melbourne, Australia; 5 University of Applied Sciences and Arts of Southern Switzerland, Switzerland; 6 Sydney School of Public Health, Faculty of Medicine and Health, University of Sydney, NSW, Australia; 7 Faculty of Medicine and Health, University of Sydney, Australia; 8 School of Population Health, UNSW, Sydney, Australia; 9 The George Institute for Global Health, New Delhi, India

**Keywords:** collaboration, cardiovascular disease, primary care, allied health, general practitioner, pharmacist

## Abstract

**Objective:**

To assess whether inter-professional, bidirectional collaboration between general practitioners (GPs) and pharmacists has an impact on improving cardiovascular risk outcomes among patients in the primary care setting. It also aimed to understand the different types of collaborative care models used.

**Study design:**

Systematic review and Hartung-Knapp-Sidik-Jonkman random effects meta-analyses of randomised control trials (RCTs) in inter-professional bidirectional collaboration between GP and pharmacists assessing a change of patient cardiovascular risk in the primary care setting.

**Data sources:**

MEDLINE, EMBASE, Cochrane, CINAHL and International Pharmaceutical Abstracts, scanned reference lists of relevant studies, hand searched key journals and key papers until August 2021.

**Data synthesis:**

Twenty-eight RCTs were identified. Collaboration was associated with significant reductions in systolic and diastolic blood pressure (23 studies, 5,620 participants) of –6.42 mmHg (95% confidence interval (95%CI) –7.99 to –4.84) and –2.33 mmHg (95%CI –3.76 to –0.91), respectively. Changes in other cardiovascular risk factors included total cholesterol (6 studies, 1,917 participants) –0.26 mmol/L (95%CI –0.49 to –0.03); low-density lipoprotein (8 studies, 1,817 participants) –0.16 mmol/L (95%CI –0.63 to 0.32); high-density lipoprotein (7 studies, 1,525 participants) 0.02 mmol/L (95%CI –0.02 to 0.07). Reduction in haemoglobin A1c (HbA1C) (10 studies, 2,025 participants), body mass index (8 studies, 1,708 participants) and smoking cessation (1 study, 132 participants) was observed with GP-pharmacist collaboration. Meta-analysis was not conducted for these changes. Various models of collaborative care included verbal communication (via phone calls or face to face), and written communication (emails, letters). We found that co-location was associated with positive changes in cardiovascular risk factors.

**Conclusion:**

Although it is clear that collaborative care is ideal compared to usual care, greater details in the description of the collaborative model of care in studies is required for a core comprehensive evaluation of the different models of collaboration.

## Introduction

Cardiovascular disease (CVD) continues to be a major contributor to mortality, morbidity and health expenditure globally [1,2]. There is a rising prevalence of CVD globally indicating the need of models of care that are patient centred and improve health outcomes [3,4,5].

Despite the availability of low-cost treatment, there remains a significant treatment gap in those with established or at high risk of CVD in the primary care setting [6,7]. This gap in the uptake of preventive measures outlines an area to focus current resources. In high income countries like Australia, 83% of Australians attend a General Practitioner (GP) every year [8]. These consultations provide opportunities to implement primary and secondary prevention measures. However, with the rise of chronic disease and maldistribution of physicians across urban and rural regions as well as the high costs involved in physician-centred models of care, new models have been suggested, including the utilisation of the skills of other health professionals such as pharmacists [9].

Pharmacists are often highly accessible healthcare professionals qualified in pharmacotherapies of diseases, identifying adherence problems, and addressing adverse drug effects, allowing them to provide valuable drug therapy recommendations to physicians. Pharmacist-based interventions alone have shown to be effective in the improvement of CVD and diabetes outcomes [10,11]. These studies have demonstrated a reduction in HbA1C in patients with diabetes [12], improvement in systolic blood pressure control and reduction in total cholesterol measurements [12,13]. Despite this evidence, pharmacists remain an underutilised member of the multidisciplinary healthcare team in the primary care setting [14].

In spite of the growing need to evaluate the use of multidisciplinary team models [15], especially in chronic disease management [16], only two published systematic reviews have investigated the effect of the inter-professional relationship between general practitioners and pharmacists on patient outcomes [17,18]. The first, found an association between the extent of collaboration and implementation rate of recommendations. The second, suggested that pharmacist interventions have a positive impact on the management of hypertension and hypercholesterolemia and the management of drug-related problems. While other reviews assessed pharmacy interventions in the primary care setting but did not focus on collaborative care between GPs and pharmacists [19,20,21]. The results of these reviews found that pharmacist intervention was associated with improvements of CVD risk factors.

Studies have found that direct discussion between pharmacist, physicians, other healthcare professionals and patients is an essential component of successful management [22]. Based on this evidence, primary care practices have hired clinical pharmacists to collaborate with physicians [23]. However, this evidence has not been synthesised. As there is no set definition for collaboration within the literature, our study is specifically focusing on bidirectional communication as collaboration. Therefore, this study aims to review current literature to assess whether GP and pharmacist collaboration improves patients’ CVD risk factors or management within the primary care setting and gain an understanding of the different types of collaborative care models used.

## Methods

The methods to this review were previously published and is outlined in brief [24]. PROSPERO registration: 2017 CRD42017055259.

### Search strategy

We used the PRISMA guideline to search MEDLINE, EMBASE, Cochrane, CINAHL and International Pharmaceutical Abstracts databases from the beginning of each database until August 2021 [25]. Further studies were obtained from scanning reference lists of relevant studies, hand searching of key journals and citation searching of key papers identified for inclusion.

Papers were independently assessed for inclusion to reduce risk of bias, and reasons for exclusion were documented. Discrepancies between two reviewers was resolved by consultation with the senior author.

### Study inclusion and exclusion criteria

This systematic review focused on randomised control trials (RCT) of inter-professional two-way collaboration between GP and pharmacists assessing a reduction of patient cardiovascular risk or improvement in CVD management in the primary care setting. Bidirectional collaboration is defined as back-and-forth communication between the GP and pharmacist. This included verbal (face to face or phone call) or written (letters, fax, email or medical note) communication. Articles were excluded if they were not a journal article, not in written in English, not a report based on empirical research (e.g. protocol, editorial), reviews and not human research.

### Data collection

Data were collected using a standardised data extraction form. The form was piloted and reviewed before being finalised. The three reviewers independently extracted data from the included articles. Data extracted included general study information as well as study, participant and intervention characteristics. Corresponding authors were contacted to obtain unpublished data or to clarify ambiguities.

### Outcomes

The primary outcome of this study was to assess the effect of GP and pharmacy collaboration on patient cardiovascular risk and CVD risk factors. Quantitative cardiovascular outcomes included blood pressure (BP), total cholesterol, low-density lipoproteins (LDL), high-density lipoproteins (HDL), triglyceride changes, Hb_A1C_, body mass index (BMI), smoking, cost effectiveness. The secondary outcome was to describe the different types of GP and pharmacist collaboration models.

### Data analysis

For all the outcomes we performed Hartung-Knapp-Sidik-Jonkman random effects meta-analysis of mean differences. We have included all studies which reported mean differences and standard deviations/errors: for the studies which had not reported standard errors we have imputed those by values predicted from the regression between observed (log) mean differences and (log) standard errors.

We assessed the quantitative heterogeneity by conducting a formal test of homogeneity and evaluating the proportion of variability due to heterogeneity (I^2^). The assessment of small-study effects has been done by regression-based Egger test and eyeball evaluation of the contour-enhanced funnel plots.

Along with the pooled effect sizes and 95% confidence intervals, we also reported the prediction intervals. All pooled results are presented in the form of forest plots. All statistical analyses were performed using Stata 17 (StataCorp LLC, College Station, TX, USA).

### Assessment of risk of bias

Risk of bias of included studies was assessed separately by two investigators using a Risk of Bias Tool. Studies were not excluded from analysis because of methodological flaws if they otherwise met inclusion criteria.

## Results

### Search and study selection

**Figure 1:**
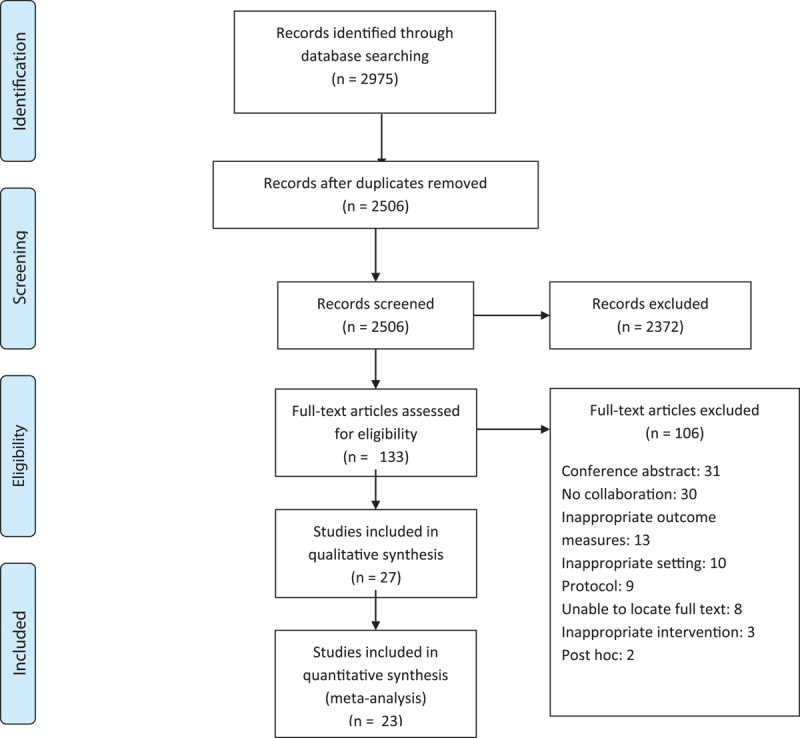
Flow chart of study retrieval and selection.

The search retrieved 2,875 potentially relevant citations. After initial screening of title and abstract, 133 full text papers were assessed of which 106 did not meet the eligibility criteria. A total of 27 studies were included in the final review (Figure 1).

### Summary of included studies

Twenty-seven RCTs representing a total of 7,442 participants were included in this review. Table 1 summarises the characteristics of included studies. Trials were published between 1973 and 2020 in peer reviewed journals. Majority of the studies were conducted in the United States of America (16/27), with the studies originating from 10 countries in total. Three cluster RCTs were included with the remaining trials randomised at patient level. While conducting the review 20 authors were contacted, 2 for additional data and 18 for clarification regarding the nature of the collaboration of which 50% (10/20) responded.

**Table 1:** Description of included studies.


AUTHOR (YEAR)	COUNTRY	CONDITION OR RISK FACTOR	OUTCOME EXTRACTED	STUDY SETTING	INTERVENTION	RECOMMENDATIONS ACCEPTED BY PHYSICIAN	METHOD OF COLLABORATION

Albsoul-Younes, 2011	Jordan	Hypertension	Change in BP	Co-location- unclear	Patient education and counselling, assessing medication regimen (noted patient medication history), adherence assessment, physical assessment.	65 (101/161)	Verbal: face to face – unclear

Aguiar, 2018	Brazil	Type 2 Diabetes	Change in BP, LDL, HbA1c	Separate	Patient education and counselling, assessing medication regimen, adherence assessment.	Not documented	Unclear

Amariles, 2012	Spain	CVD	Change in BP, TC	Separate- unclear	Patient education and counselling, assessing medication regimen, physical assessment.	Not documented	Unclear

Anderegg, 2018	United States of America	Type 2 Diabetes and Chronic Kidney Disease	Change in BP	Co-location	Patient education and counselling, assessing medication regimen, individualised care plan.	Not documented	Verbal or by electronic communication

Bogden, 1997	United States of America	Hypercholesterolemia	Change in TC	Co-location	Patient education and counselling, assessing medication regimen, physical assessment (were done but not clear if it was by pharmacist).	90 (167/186)	Verbal: face to face – unclear

Bogden, 1998	United States of America	Hypertension	Change in BP	Co-location	Patient education and counselling, assessing medication regimen, physical assessment (were done but not clear if it was by pharmacist).	93 (150/162)	Verbal: face to face – unclear

Borenstein, 2003	United States of America	Hypertension	Change in BP	Separate	Patient education and counselling, assessing medication regimen, adherence assessment, physical assessment.	Not documented	Verbal: phone

Carter, 1997	United States of America	Hypertension	Change in BP	Separate but in the same building	Patient education and counselling, assessing medication regimen, adherence assessment. (questioned about adherence), physical assessment.	Not documented	Verbal: mostly face to face but some over the phone (immediate) and written feedback too

Carter, 2008	United States of America	Hypertension	Change in BP	Co-location	Patient education and counselling, assessing medication regimen, adherence assessment, (some, not all allowed) independent prescribing, physical assessment.	96 (256/267)	Verbal: face to face

Carter, 2009	United States of America	Hypertension	Change in BP	Co-location	Assessing medication regimen, physical assessment.	96 (742/771)	Verbal: face to face

Carter, 2015	United States of America	Hypertension	Change in BP	Co-location	Patient education and counselling, assessing medication regimen, adherence assessment, physical assessment.	Not documented	Mostly verbal: face to face, some written: email

Choe, 2005	United States of America	Type 2 diabetes	Change in HbA1C	Co-location	Patient education and counselling, assessing medication regimen, physical assessment (upon need).	Not documented	Verbal: face to face

Ebid, 2020	Egypt	Type 2 Diabetes	Change in LDL, HDL, HbA1c, BMI	Co-location	Patient education and counselling, assessing medication regimen, physical assessment, adherence assessment.	Not documented	Unclear

Fornos, 2006	Spain	Type 2 diabetes	Change in BP, TC, LDL, HDL, TRIG, HbA1C, BMI	Separate	Patient education and counselling, assessing medication regimen, physical assessment, adherence assessment.	Not documented	Unclear

Geurts, 2016	Netherlands	CVD	Change in BP, TC, LDL, HDL, HbA1C, BMI	Separate	Assessing medication regimen, physical assessment.	Not documented	Written: webbased pharmaceutical care plan tool

Hammad, 2011	Jordan	Metabolic syndrome	Change in BP, HDL, TRIG, WEIGHT AND CM	Co-location	Patient education and counselling, assessing medication regimen, adherence assessment (?), physical assessment.	70 (128/182)	Verbal

Hirsch, 2014	United States of America	Hypertension	Change in BP, LDL, HDL	Co-location	Patient education and counselling, assessing medication regimen (noted patient medication history) adherence assessment, physical assessment, independent prescribing.	Not documented	physician was always present in the medical practice during the pharmacist clinic visits and was available for consultation as needed.

Hunt, 2008	United States of America	Hypertension	Change in BP	Co-location	Patient education and counselling, assessing medication regimen, adherence assessment, physical assessment, independent prescribing.	Not documented	Written: emr (note documented and forwarded to PCP for approval and signature, verbal: *face to face – not clarified* (if needed)

McKenney, 1973	United States of America	Hypertension	Change in BP	Separate	Patient education and counselling, assessing medication regimen, adherence assessment, physical assessment.	100 (37/37)	written and verbal

Odegard, 2005	United States of America	Type 2 diabetes	Change in HbA1C	Co-location	Patient education and counselling (?), assessing medication regimen, adherence assessment, physical assessment (?).	Not documented	EMR

Rothman, 2005	United States of America	Type 2 diabetes (poorly controlled)	Change in BP, TC, HbA1C, WEIGHT	Co-location	Patient education and counselling, assessing medication regimen, physical assessment, independent prescribing (with approval of PCP).	Not documented	Written (results shared and written approval for medication change) & verbal: face to face (could be in the consult with the physician) or phone (could call them for approval)

Scott, 2006	United States of America	Type 2 diabetes	Change in BP, LDL, HDL, HbA1C, WEIGHT AND BMI	Co-location	Patient education and counselling, assessing medication regimen, adherence assessment (?), physical assessment, independent prescribing (?).	Not documented	not specified

Siaw, 2017	Singapore	Type 2 Diabetes	Change in BP, LDL, HbA1c, costs	Co-location	Patient education and counselling, assessing medication regimen, physical assessment, optimising medication dosing and frequency.	Not documented	Written

Simpson, 2011	Canada	Type 2 diabetes patients with hypertension	Change in BP, TC, LDL, HDL, TRIG, HbA1C, BMI	Co-location	Patient education and counselling, assessing medication regimen, adherence assessment, physical assessment.	Not documented	Verbal

Sookaneknun, 2004	Thailand	Hypertension	Change in BP	Separate	Patient education and counselling, assessing medication regimen, adherence assessment, physical assessment.	47 (96/206)	Written: letter and medical record note

Tobari, 2010	Japan	Hypertension	Change in BP, BMI, SMOKING CESSATION	Co-location	Patient education and counselling, assessing medication regimen adherence assessment, physical assessment.	Not documented	EMR and verbal (if necessary): phone or face to face

Zillich, 2005	United States of America	Uncontrolled hypertension	Change in BP	Separate	Patient education and counselling, assessing medication regimen, adherence assessment, physical assessment.	75 (43/57)	Written: EMR and verbal: phone


### Primary outcome

**Figure 2:**
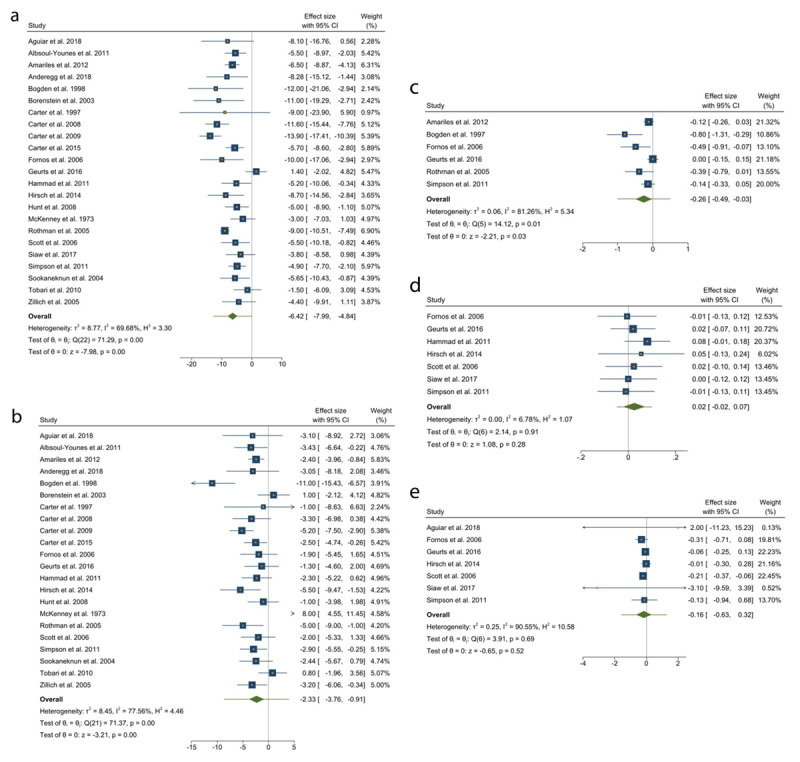
Random effects meta-analysis of the change in cardiovascular risk factors (systolic blood pressure, diastolic blood pressure, total cholesterol, low density lipoproteins, high density lipoproteins) based on collaboration of general practitioner and pharmacist or usual care. **a.** Systolic blood pressure. **b.** Diastolic blood pressure. **c.** Total cholesterol. **d.** High density lipoproteins. **e.** Low density lipoproteins.

**Figure 3:**
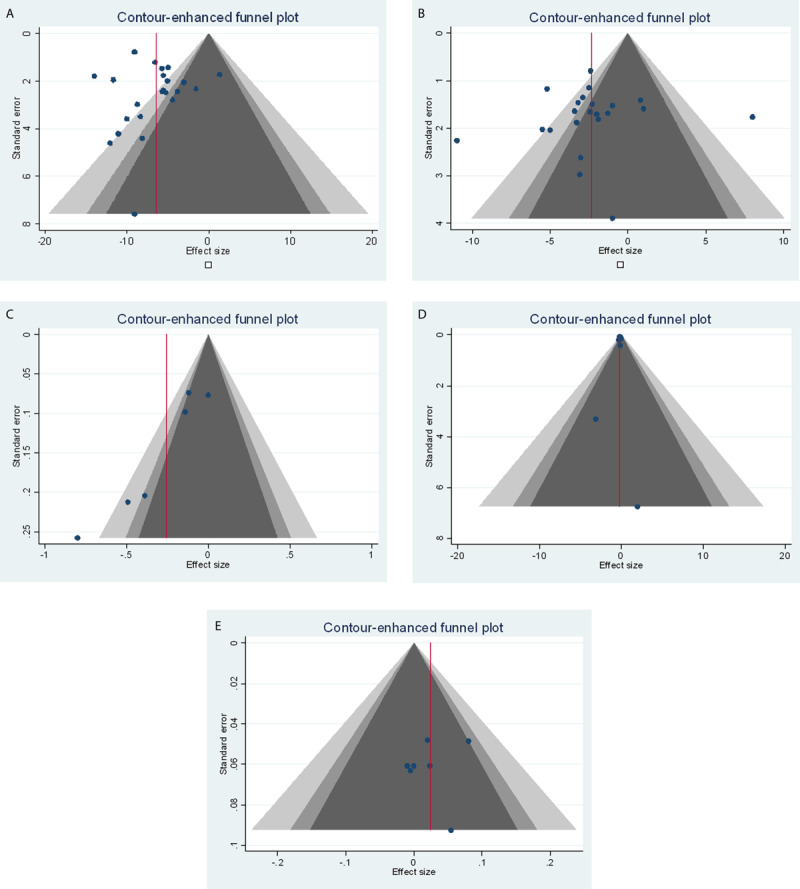
Contour enhanced funnel plot for random effects meta-analysis of the change in cardiovascular risk factors (systolic blood pressure, diastolic blood pressure, total cholesterol, low density lipoproteins, high density lipoproteins) based on collaboration of general practitioner and pharmacist or usual care. **a.** Systolic blood pressure. **b.** Diastolic blood pressure. **c.** Total cholesterol. **d.** Low density lipoproteins. **e.** High density lipoproteins.

BP was measured as an outcome in 23 studies (7,082 participants). Intervention resulted in a reduction in SBP and DBP outcome in all studies. Twenty-three RCT’s were included in the meta-analysis for SBP and 22 for DBP. Overall, collaboration in addition to medication titration was associated with a reduction in SBP and DBP of –6.42 mmHg (95%CI –7.99 to –4.84, Figure 2a) and –2.33 mmHg (95%CI –3.76 to –0.91, Figure 2b), respectively. Regression-based Egger test for small-study effects random-effects model found no publication bias in included studies measuring SBP and DBP (Figure 3a and 3b, respectively).

Twelve studies measured changes in cholesterol. Changes were measured in total cholesterol (6 studies, 1,917 participants), LDL (8 studies, 1,817 participants), HDL (7 studies, 1,525 participants) and triglycerides (3 studies, 573 participants). Four studies were included in the meta-analysis for changes in total cholesterol which was associated with a reduction of –0.26 mmol/L (95%CI –0.49 to –0.03, Figure 2c). Four studies were included in the meta-analysis for LDL which was associated with a reduction of –0.16 mmol/L (95%CI –0.63 to 0.32, Figure 2d). Four studies were included in the meta- analysis for HDL which was associated with an increase of 0.02 mmol/L (95%CI –0.02 to 0.07, Figure 2e). Regression-based Egger test for small-study effects random-effects model found statistically significant publication bias (Figure 3c, p = 0.0015), however this analysis only included six studies. Furthermore, no publication bias was observed in included studies measuring LDL and HDL (Figure 3d and 3e, respectively).

Other studies measured changes in HbA1C (10 studies, 2,025 participants), BMI (8 studies, 1,708 participants) and smoking cessation (1 study, 132 participants). Mean difference in HbA1C was measured as a percentage or mmol/mol. The change was –0.4 (–1.6 to 0.7%) in the control to –1.2% (–2.5% to –0.15%) in the intervention group. Reduction in BMI was greater in the intervention group with BMI mean reduction –0.5 (–0.3 to –0.9) compared to a mean reduction of –0.04 (–0.3 to 0.5) in the control. One study assessed smoking cessation and found that two of the participants in the control group ceased smoking compared to eight participants in the intervention group.

None of the included studies addressed cost effectiveness. However, three studies assessed the changes in cost of the drug and visit which were all calculated in United States Dollars but were measured differently. Cost of medication and visits varied in the intervention compared to the control group. One study reported visit cost per patient ($160 vs $195) was lower, but drug costs ($11.31 vs $4.25) from baseline was higher in the intervention compared to the control group. Another found that mean drug ($317 vs $212) and visit charges ($823 vs $336) for the intervention group was higher control group. The final study found that the medication ($319 vs $410) charges was lower but visit ($115 vs $112) charges were greater in the intervention compared to control group.

### Secondary outcome

Participants were followed over a median of eight months (range 3–12 months). The interventions have been summarised in Table 1. They involved (1) patient education and counselling (3%, 25/27); (2) assessing medication regimen (100%, 27/27); (3) adherence assessment (can involve implementation of reminder systems) (67%, 18/27); and (4) physical assessments (i.e., measuring BP) (93%, 25/27).

Only one study specified the doctor as a GP with the remaining studies referring to them as physicians (70%, 19/27) or primary care physicians (26%, 7/27). Collaboration between the pharmacist and physician was undertaken in two main settings; co-location (56%, 15/27) and separate (30%, 8/27) locations and some were not specified (15%, 4/27). Co-location was associated with positive changes within CVD risk factors. Inter-professional communication was either purely verbal (face-to-face or over the phone) (33%, 9/27), purely written (letter or electronic) (19%, 5/27), a combination of the two (26%, 7/27) or not specified (22%, 6/27).

Nine out of 28 studies recorded the number of recommendations made by the pharmacist. Across the studies, 2,029 recommendations were made by the pharmacist, of these 85% (1720/2029) were accepted by the physician.

### Methodological quality of studies

A summary of the proportion of trials that were at low, unclear, and high bias for each domain is shown in Supplementary File 1. Among twenty-eight RCT most domains remained low risk of bias. Information about selective reporting was unclear for most trials. Allocation concealment was also unclear for approximately half the trials.

## Discussion

Our research identified 28 RCT’s that evaluated collaboration between GP and pharmacists. Our meta-analysis found that collaborative care achieved reduction in BP and cholesterol, however reduction in cholesterol was modest. Reduction in HbA1C and BMI as well as increasing smoking cessation was also noted with collaborative care. In the three studies that evaluated costs, this model of care was associated with increased costs. The most frequent form of collaboration provided was assessing medication regime and performing regular physical measurements. Our findings are consistent with previous similar reviews which suggest that pharmacist interventions have a positive impact on the management of cardiovascular risk factors [18,20]. However, these reviews have not focused on bidirectional collaboration, rather just pharmacist intervention in general and have not specified the nature of GP involvement. This meta-analyses found similar changes to CVD risk factors to other reviews however it was difficult to differentiate which GP and pharmacist model attributed to the changes due to the lack of information provided within the studies [20]. Additionally, included studies found improvement in CVD risk factors within the control group. However, this may be attributed to over half the studies having the pharmacist and physician co-located which may have led to cross contamination of the groups. Furthermore, the fact that study participants tend to be healthier than non-participants and the Hawthorne effect could have also had an impact on improvements within the control group [23,26,27]. The Hawthorne effect has been shown to improve adherence to medications which may result in improvements to cardiovascular risk factors such as BP. These influences are worthy of further exploration, nevertheless collaboration between pharmacist and physician resulted in favourable changes in cardiovascular risk factors. To our knowledge this was the first review to evaluate bidirectional collaboration between GP and pharmacist, rather than a pharmacist or GP led intervention management.

Lifestyle modifications, like weight control and smoking cessation, are important risk factors that can affect cardiovascular outcomes. Proportion of participants who quit smoking was significantly larger in the intervention group than in the control group, however was only assessed by one study [28]. BMI was generally lower in those who received collaborative care, however the change in BMI still minimal and similar to those who were within the control group. Interdisciplinary collaboration could lead to more effective lifestyle modifications not confined to weight and smoking, such as reduction in sodium, which could be effective in hypertension control and potentially allow for reduction in antihypertensive medications [28]. Preventive measures, such as aspirin therapy and influenza vaccination, are also more prevalent in those who received collaborative care [23].

Overall, costs of medications and visits was varied, however no cost effect analysis was conducted. This could suggest that the interventions were potentially more effective in increasing uptake in drugs in those that were under-treated. Furthermore, mean costs per visit lower in the intervention group and could be attributed to a lower average number of visits to the primary care [29]. Nonetheless, it is evident that more visits are associated with increased collaboration [29,30]. This may reflect more effective follow up in patients who undergo collaborative care. However, these costs could be minimised if the physician-pharmacist collaboration occurs over the phone at the time of the visit by the patient to the physician’s office [28]. The increased cost of medications, although not ideal, could offset other savings associated with poor health outcomes. These include downstream healthcare costs of worsening CVD risk factors can lead to increased costs by hospitalisations, more medications, and visits with specialists.

Informal in-person discussions have been found to facilitate richer exchange of patient specific ideas compared to impersonal emails or faxes that are more commonly used [22,27]. This model of care is pragmatic, people centred and accessible, however is not often used which is evident as only a third of the included studies reported communicating verbally. These informal discussions may be more achievable through a co-located pharmacy which can further facilitate collaborative care. Although over half the studies included were co-located, method of collaboration vastly varied. This may be attributed to the lack of clear definition of collaboration, with each individual having their own unique understanding. Other studies have found that majority of participants state that they do not receive public health services from pharmacies and do not expect them, yet those who have received them have been more satisfied [31]. It is evident that participants who receive collaborative care report higher levels of satisfaction, were less worried about their health and had a higher perceived health level than usual care [23]. This model of care requires a good relationship as well as trust among the collaborators and a shared value of improving patient outcomes. Nonetheless, pharmacists find it difficult to set up cooperation potentially due to a lack of time or premises to convince the GP to cooperate [23]. It is evident that further research needs to be undertaken to understand the barriers and facilitators of collaborative care and the adaption of this model. Additionally, other models of integrated care that involve GPs and nurses or nurse practitioners as well as other allied health including dietitians, diabetes educators and community care workers need to be considered [32,33].

### Limitations

Our review had some limitations. Firstly, collaboration was defined as bidirectional which was difficult to attain from the limited description of the intervention provided in some studies. Although authors were contacted for clarification, when no response was received it was left up to our judgement from the information provided within the study. As a result, some relevant studies may have been excluded from the review. Due to the multifaceted nature of this intervention, it was challenging to attribute the observed differences to a specific component and precisely identify which component of the intervention was more effective. There is a possibility of publication bias as not all trials are published especially when their results are unfavourable. We have included contour enhanced funnel plot (Figure 3) to indicate evidence of publication bias specifically in Figure 3c measuring total cholesterol. It is important to note that only included six studies. Finally, meta-analysis was not conducted for changes in HbA1C, BMI and smoking cessation.

## Conclusion

In conclusion, our meta-analysis found that bidirectional collaboration leads to improved CVD risk factors for patients. There are various models of collaborative care, however it is unclear if superiority exists. This review focused on CVD risk factors rather than longer-term clinical end points such as stroke or death. Further research is required to assess the cost-effectiveness and longer-term clinical endpoints associated with collaborative care as well as the barriers and facilitators of collaborative care.

## Additional File

The additional file for this article can be found as follows:

10.5334/gh.1184.s1 Supplementary File 1. Review authors’ judgments about each risk of bias item, presented as percentages across all included studies.
