# Supplementary material for: Does Collaboration between General Practitioners and Pharmacists Improve Risk Factors for Cardiovascular Disease and Diabetes? A Systematic Review and Meta-Analysis
Source: Glob Heart. 2023 Feb 23;18(1):7. doi: 10.5334/gh.1184 (PMC9951619; doi:10.5334/gh.1184)

Supplementary File 1. Review authors' judgments about each risk of bias item, presented as percentages across all included studies.

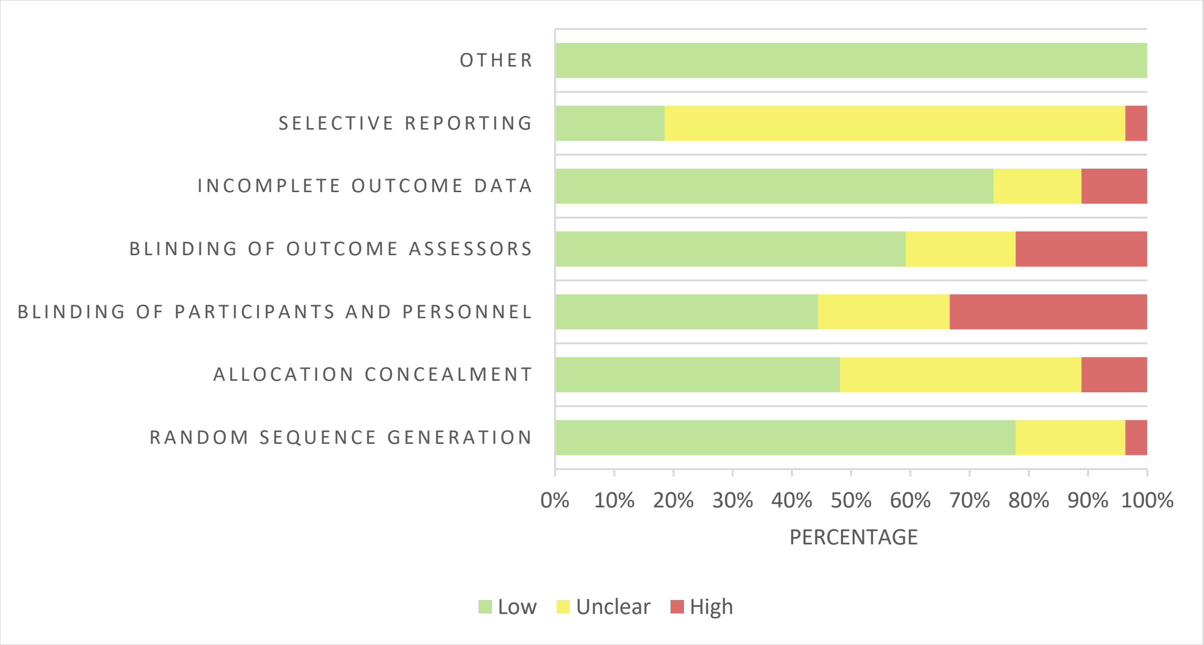

Supplement: Supplementary File 1. — Review authors’ judgments about each risk of bias item, presented as percentages across all included studies. [file gh-18-1-1184-s1.pdf]
